# Modulating Influence of State Anxiety on the Effect of Midazolam on Postsurgical Pain

**DOI:** 10.3390/jcm12072669

**Published:** 2023-04-03

**Authors:** Caroline Dahlem, Catarina Monteiro, Eunice Mendes, Joana Martinho, Carmen Oliveira, Margarida Bettencourt, Miguel Coelho, Paula Neves, Luís Azevedo, Cristina Granja

**Affiliations:** 1 CINTESIS@RISE—Center for Health Technology and Services Research, Faculty of Medicine, University of Porto, 4200-319 Porto, Portugal; carmen.oliveira@chvng.min-saude.pt (C.O.); lazevedo@med.up.pt (L.A.); cgranja@med.up.pt (C.G.); 2 Department of Anesthesiology, Centro Hospitalar Entre Douro e Vouga, 4520-211 Santa Maria da Feira, Portugal; 3 Department of Anesthesiology, Centro Hospitalar de Vila Nova de Gaia/Espinho, 4434-502 Vila Nova de Gaia, Portugal; 07895@chvng.min-saude.pt (C.M.);; 4 Department of Anesthesiology, Centro Hospitalar e Universitário de Coimbra, 3004-561 Coimbra, Portugal; 11458@chuc.min-saude.pt; 5 Department of Rheumatology, Hospital de Santa Maria, Centro Hospitalar Universitário Lisboa Norte, 1649-028 Lisboa, Portugal; 6 Department of Anesthesiology, Centro Hospitalar do Baixo Vouga, EPE, 3810-164 Aveiro, Portugal; 18289@chbv.min-saude.pt (M.B.); miguel.apc90@gmail.com (M.C.); 7 Department of Community Medicine, Information and Health Decision Sciences (MEDCIDS), Faculty of Medicine, University of Porto, 4200-319 Porto, Portugal; 8 Department of Anesthesiology, Centro Hospitalar Universitário de São João, 4200-319 Porto, Portugal; 9 Department of Surgery and Physiology, Faculty of Medicine, University of Porto, 4200-319 Porto, Portugal

**Keywords:** midazolam, anxiety, postsurgical pain, inguinal hernia, anesthesia

## Abstract

Anxiety contributes to postsurgical pain, and midazolam is frequently prescribed preoperatively. Conflicting results have been described concerning the impact of midazolam on pain. This study aims to evaluate the effect of systemic midazolam on pain after open inguinal hernia repair, clarifying its relationship with preoperative anxiety. A prospective observational cohort study was conducted in three Portuguese ambulatory units between September 2018 and March 2020. Variable doses of midazolam were administered. Postsurgical pain was evaluated up to three months after surgery. We enrolled 306 patients and analyzed 281 patients. The mean preoperative anxiety Numeric Rating Scale score was 4 (3) and the mean Surgical Fear Questionnaire score was 22 (16); the mean midazolam dose was 1.7 (1.1) mg with no correlation to preoperative anxiety scores. Pain ≥4 was present in 67% of patients 24 h after surgery and in 54% at seven days; at three months, 27% were classified as having chronic postsurgical pain. Preoperative anxiety correlated to pain severity at all time points. In multivariable regression, higher midazolam doses were associated with less pain during the first week, with no apparent effect on chronic pain. However, subgroup analyses uncovered an effect modification according to preoperative anxiety: the decrease in acute pain occurred in the low-anxiety patients with no effect on the high-anxiety group. Inversely, there was an increase in chronic postsurgical pain in the very anxious patients, without any effect on the low-anxiety group. Midazolam, generally used as an anxiolytic, might impact distinctively on pain depending on anxiety.

## 1. Introduction

Anxiety is reported by most patients as the worst aspect of the perioperative period [1]. Excessive anxiety is associated with increased postsurgical pain and decreased patient satisfaction [2,3,4]. Formal anxiety evaluation is not part of standard preanesthetic assessment, but anesthesiologists routinely prescribe benzodiazepines, especially midazolam, as anxiolytic preoperative medication. Current evidence indicates that midazolam premedication does not significantly reduce preoperative anxiety [5].

Midazolam is a γ-aminobutyric acid type A (GABA A) receptor modulator, binding to a specific benzodiazepine site. It is popular due to combining anxiolytic, sedative, hypnotic, anticonvulsant, muscle relaxant, and anti-emetic properties with rapid onset, short half-life and good safety profile [6,7,8]. The spinal administration of midazolam is known to provide analgesia [8]. However, systemic administration has resulted in hyperalgesic effects in both rats [9] and humans [6,10,11]. Moreover, the evidence is conflicting, with a few other human studies claiming that systemic midazolam is analgesic [7,12,13], while others found no effect on pain [14,15,16,17].

A possible explanation for such inconsistent results might be that different timings and/or surrogates of pain were used or that anxiety could have confounded or modified this effect. Acute pain is influenced by multiple endogenous, physical, and emotional factors. State anxiety is indeed one of the most significant predictors of postsurgical pain [2].

Following previous results, where we found that midazolam was associated to increased postsurgical pain in ambulatory knee arthroscopy [11], we hypothesized that midazolam administered preoperatively might increase postsurgical pain perception. Therefore, the main objective of this study was to evaluate whether systemic midazolam had any effect on postsurgical pain following open inguinal hernia repair. The secondary goal was to clarify the relation between preoperative anxiety, midazolam, and postsurgical pain.

## 2. Materials and Methods

This multicentre prospective observational cohort study was preregistered prior to patient enrollment at clinicaltrials.gov accessed on 17 April 2018 (NCT03499730) by principal investigator Caroline Dahlem. Ethical approval for this study was provided by the Ethical Committees of Centro Hospitalar Vila Nova de Gaia/Espinho, Vila Nova de Gaia; Centro Hospitalar do Baixo Vouga, Aveiro; and Centro Hospitalar Entre Douro e Vouga, Santa Maria da Feira, Portugal. Written informed consent was obtained from all study participants. This manuscript adheres to the applicable STROBE guidelines.

This multicentric prospective observational cohort study was registered at clinicaltrials.gov accessed on 17 April 2018 (NCT03499730).

### 2.1. Participants

Setting: three ambulatory surgery units serving different populations from Portugal; patients were recruited between 12 September 2018 and 5 March 2020.

Inclusion criteria: adult patients scheduled for open inguinal hernia repair who agreed to participate and sign an informed consent form. Exclusion criteria included psychiatric disorders or alcoholism; illiteracy or a poor understanding of the Portuguese language; a history of chronic pain under opioid treatment; recurrent hernia; and simultaneous bilateral surgery.

We chose to study patients submitted to open inguinal hernia repair, as this is a common ambulatory surgical procedure and most patients experience both acute and chronic postsurgical pain [18].

### 2.2. Protocol

All patients had a standard preoperative assessment with an anesthesiologist several weeks before surgery. Before arrival at the operating room, patients received preoperative medication: 10 mg of metoclopramide, 40 mg of esomeprazole, and variable doses of intravenous (IV) midazolam. The attending anesthesiologist would define the midazolam dosage according to his own criteria (from none to any dosage) as well as the anesthesia protocol (general or spinal). General anesthesia was induced with 2–2.5 μg.Kg^−1^ of fentanyl and 1–2 mg.Kg^−1^ of propofol, and maintained with sevoflurane through a laryngeal mask. Anti-emetics were administered according to risk factors. Spinal anesthesia was performed with 8–9 mg of 0.5% hyperbaric bupivacaine in lateral decubitus. At the end of surgery, all patients received local wound infiltration with 10 mL of ropivacaíne 0.75%, 1 g of IV acetaminophen, and 30 mg of ketorolac, and rescue analgesia was given in the recovery room with 2 mg.Kg^−1^ of tramadol. All patients received take-home oral analgesia: 1 g of acetaminophen (6/6 h), 400 mg of ibuprofen (8/8 h), and 50 mg of rescue tramadol (8/8 h). If there was an intolerance or allergy, ketorolac and/or ibuprofen were replaced by 2 g of metamizole. All centers used a polypropylene Bard^®^ Mesh Perfix Plug for surgery (Davol Inc, Providence, RI, USA).

### 2.3. Variables

The predictor variable was the total IV midazolam dose in mg. The primary outcome was postsurgical pain evaluated by an 11-point numeric rating scale (NRS) (0–10) by telephone interview at three different follow-up points: 24 h, 7 days, and 3 months after surgery. Secondary outcomes included: total analgesic consumption at 24 h and 7 days; adverse events; patient satisfaction; and Global Surgery Recovery Index (GSRI) scores. We considered preoperative anxiety as the main confounder because it might interfere with both midazolam dosage and postsurgical pain. Other variables that considered potential confounders were preoperative pain, sex, ambulatory unit, chronic benzodiazepine consumption, smoking, and the type of anesthesia. Chronic postsurgical pain (CPSP) was defined according to ICD-11 as pain that developed or increased in intensity after surgery and persisted for at least 3 months [19].

### 2.4. Data Collection

Age, weight, height, sex, smoking status, employment status, education level, preoperative pain, and anxiety were assessed by a self-reported preoperative questionnaire on the day of surgery, supported when needed by one of the study investigators. Preoperative anxiety was assessed both as an 11-point NRS, from 0 to 10, and via the Surgical Fear Questionnaire (SFQ), validated for the Portuguese population. It consists of two subscales: fear of the short- and long-term consequences of surgery [20]. A simple Visual Analog Scale has been proven to be a fast, easy, and reliable tool for evaluating state anxiety and has been validated in the preoperative context [21]. Preoperative pain was assessed using an 11-point NRS (0–10) and the Pain Severity Scale from the Portuguese validated version of the Brief Pain Inventory [22]. Perioperative data were recorded in theatre and included American Society of Anesthesiologists physical status (ASA), chronic benzodiazepine use, surgical and anesthetic techniques, midazolam dose, and postoperative analgesia. Follow-up telephone interviews were carried out by two blind investigators. Data included pain NRS (at rest, movement-evoked, average), analgesic consumption, patient satisfaction NRS (0–10), adverse events, and GSRI scores (0–100).

### 2.5. Study Size

The sample size was calculated considering a power of 80%, α = 0.05, a mean 24 h postsurgical pain NRS of 4.5 (1.5) [23], and an attrition of 15%; we would need at least 300 patients to detect clinically relevant differences of 1.0 or higher.

### 2.6. Statistical Analysis

Statistical analyses were performed using IBM^®^ SPSS^®^ (version 26; IBM Company, Armonk, NY, USA) considering a significance level of *p* = 0.05. Categorical variables are presented as *n* (%) and compared using the Pearson chi-square test. Pain and anxiety scores were considered continuous variables. Continuous variables were considered normally distributed when skewness and kurtosis varied between −0.7 and 0.7; these data are presented as mean (SD) values and compared using Student’s *t*-test or one-way ANOVA. Other continuous variables are presented as median [IQR]. All tests are two-sided and referenced in tables. Missing data were not replaced and outlying data were not removed. When appropriate, age, body mass index (BMI), and pain and anxiety scores were transformed into categorical variables. Preoperative pain was defined as NRS ≥ 4 and significant postsurgical pain was defined as NRS ≥ 4. For age and SFQ anxiety scores, groups were defined using the terciles; for BMI, the mean value was used. Pearson correlation was used to assess the association between anxiety scales, postsurgical pain, midazolam dosage, and analgesic consumption. Due to the uneven administration of midazolam among some groups of patients, the effect of midazolam on pain was searched for through multivariable logistic regression models. Preoperative pain, anxiety SFQ, sex, age, ambulatory unit, and the type of anesthesia were forced into all logistic models, as they were considered major potential confounders due to possible influence on both midazolam dose and postsurgical pain. In the models, we also included variables that were significantly associated with the pain outcome variables (if *p* < 0.1). Multicollinearity was assessed in all models; all predictor variables had a variance inflation factor < 2. Estimates of adjusted odds ratios (adj ORs) with 95% confidence intervals (95% CI) are presented. While running these logistic regression models, an interaction of midazolam with preoperative anxiety was significant for some pain outcomes. To clarify this interaction, we ran the same models separately on the anxiety subgroups, defined by the terciles of the SFQ scores. We also explored subgroups and effect modification according to sex, the type of anesthesia, and the ambulatory unit.

## 3. Results

### 3.1. Participants

**Figure 1:**
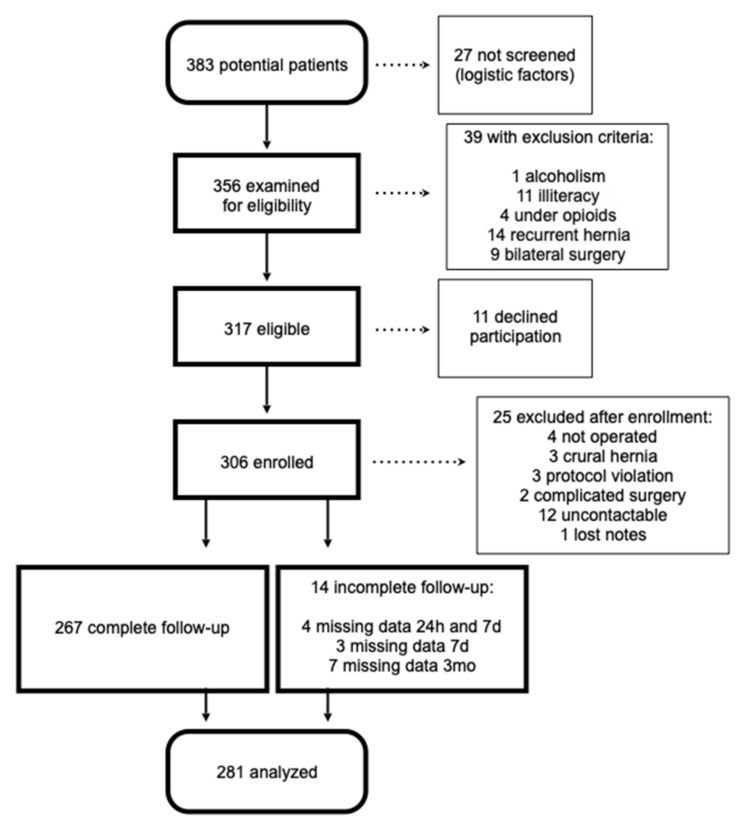
STROBE diagram of patient recruitment.

Three hundred and six patients were enrolled between 12 September 2018 and 5 March 2020; unit 3 stopped enrollment earlier than planned due to SARS-CoV-2 pandemic restrictions. A total of 267 patients completed the three months follow-up and 281 were analyzed. The study flow chart is presented in Figure 1.

In total, 31 (11%) women and 250 (89%) men aged 57 (13) years old were included in the analysis. The mean BMI was 25.6 (2.8) and most patients (97%) had an ASA status < 3. The preoperative anxiety NRS was 4 (3) and the SFQ was 22 (16); there was a significant positive correlation between the two measures (r = 0.522 [0.419 to 0.622], *p* < 0.001). The median preoperative pain NRS was 1 [3] and a midazolam dose of 1.7 (1.1) mg or 23.7 (14.4) μg.Kg^−1^ was administered. Relevant characteristics and perioperative data are presented in Table 1.

**Figure 2:**
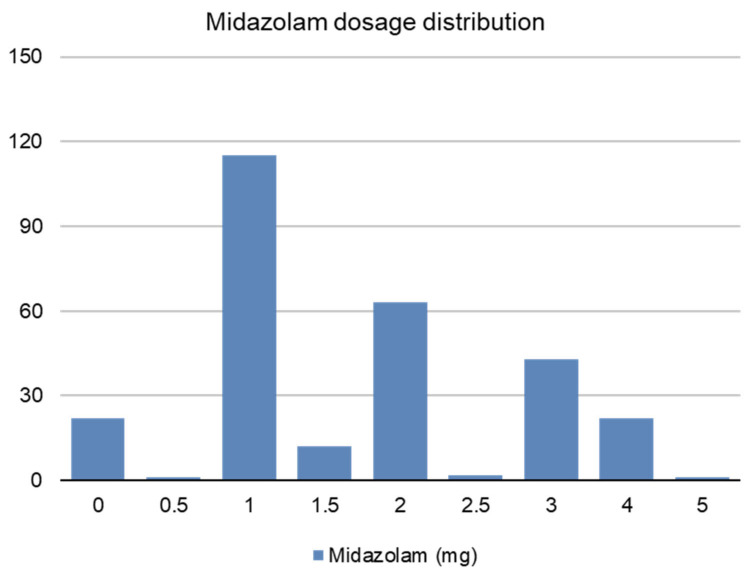
Midazolam dosage distribution.

Midazolam dosage distribution is presented in Figure 2, and was not correlated to preoperative anxiety (*r* = 0.040 [−0.079 to 0.157], *p* = 0.511). Patients under spinal anesthesia received twice as much midazolam as patients under general anesthesia: 2.4 (1.1) vs. 1.1 (0.7) mg, or 32.0 (14.5) vs. 15.9 (9.1) μg.Kg^−1^, *p* < 0.001.

Twenty-three patients had anesthetic complications: seventeen with postoperative nausea and vomiting (four spinal/thirteen general anesthesia) and six with headaches (two spinal/four general anesthesia). Patients under general anesthesia needed rescue analgesics more frequently in the post-anesthesia care unit than the ones receiving spinal anesthesia: 64% vs. 21% (*p* < 0.001), respectively.

### 3.2. Outcome Data

Moderate-to-severe pain was present in 67% of patients 24 h after surgery and in 54% of patients at seven days; on the first postoperative day, 11% needed rescue analgesics more frequently if they operated under general rather than spinal anesthesia (18% vs. 7%, *p* = 0.031); after the first week, 22% of patients were still on analgesics, with no difference between anesthetic techniques. The persistence of inguinal pain was present in 56% of patients at three months, but only 27% would classify as CPSP and 4% were on analgesics. Outcome data are presented in Table 2. Preoperative anxiety correlated to pain severity at 24 h (*r* = 0.299 [0.186 to 0.416], *p* < 0.001), 7 days (*r* = 0.250 [0.134 to 0.369], *p* < 0.001), and 3 months (*r* = 0.165 [0.047 to 0.285], *p* = 0.007). Analgesic consumption at 24 h and 7 days did not consistently correlate with pain intensity at those time points but was significantly different across surgical units (*p* < 0.001).

### 3.3. Impact of Midazolam on Postsurgical Pain

In multivariable logistic regression models, midazolam was associated with decreased acute pain during the first week; for each mg of midazolam, the odds of reporting significant pain at 24 h decreased by 0.630 (0.427–0.930) and any pain at 7 days decreased by 0.418 (0.175–0.997). Midazolam did not seem to influence chronic pain in the overall analysis. However, subgroup analysis suggests that the positive effect of midazolam on pain only benefitted the low-anxiety patients. If SFQ < 12, for each mg of midazolam, the odds of reporting significant pain at seven days decreased by 0.289 (0.117–0.713). On the contrary, midazolam was associated with increased CPSP exclusively in the highly anxious patients; if SFQ > 28, for each mg of midazolam, the odds of developing CPSP at three months increased by 2.124 (1.026–4.397). Results of the logistic regression models are presented in Table 3.

## 4. Discussion

In this study, midazolam was associated with decreased acute pain after ambulatory open inguinal hernia repair; each mg of midazolam significantly reduced the likelihood of reporting pain in the first week. This analgesic effect of midazolam has been described by other authors [7,12,13]. However, subgroup analyses uncovered an effect modification of midazolam according to preoperative anxiety: the decrease in acute pain occurred only in the low-anxiety patients and no effect was seen in the high-anxiety group.

On the other hand, midazolam was associated with increased CPSP, but only in the high-anxiety patients; each mg of midazolam doubled the odds of CPSP at three months, without any effect on the low-anxiety group. To our knowledge, no previous study has addressed the effect of midazolam on chronic pain and its effect modification dependent on preoperative anxiety.

This effect modification is consistent across several outcomes and cut-offs for anxiety and is also present whether we use the SFQ or the NRS to measure anxiety. However, subgroup analysis implied a loss of statistical power, which might be why we failed to find a significant effect of midazolam on acute pain in high-anxiety patients or on CPSP in low-anxiety patients. Kain and colleagues were the only authors to previously explore the effect of midazolam on pain, separately analyzing the subgroups based on trait anxiety; however, they did not find any significant effect of midazolam on pain up to one month after surgery [15].

Increased pain perception in volunteers sedated with midazolam has been previously described [10]. We have also previously found an association of midazolam with increased 24 h pain in men submitted to knee arthroscopy under spinal anesthesia [11], but in both studies anxiety was not measured and could have modified or confounded the results. Some authors have claimed that systemic midazolam is antinociceptive, using the decreased analgesic requirement as a surrogate during early postoperative hours while patients were still under high doses of midazolam (up to 90 μg.Kg^−1^) [7,13]. Again, anxiety was not evaluated in these studies and excessive sedation could per se delay the need for rescue analgesia; on the other hand, patients in the midazolam group consumed less ibuprofen during the next several days [7,13].

The major strengths of our study are a 3-month follow-up and the separate analyses by subgroups of state anxiety. This scrutiny of data clarified that anxiety appears as an effect modifier of the impact of midazolam on postsurgical pain. Though anxiety is a known risk factor for postsurgical pain, it is unclear how it mediates the effect of midazolam.

The answer might be in the GABA receptors, about which knowledge is continuously being updated. GABA A receptors are the major inhibitory neurotransmitter receptors in the human brain, being involved in inhibitory pain pathways. Midazolam modulates GABA A activity, facilitating channel opening, but different receptor subtypes produce different clinical effects [24]. Midazolam is assumed to bind to the high-affinity benzodiazepine binding site in the GABA A receptor, acting as a positive allosteric modulator. However, multiple non-canonical low-affinity sites have been described at extracellular and transmembrane locations [25]. Furthermore, it is not guaranteed that midazolam will consistently act as a positive modulator; flumazenil has been described to act as a weak negative allosteric modulator at low concentrations and as a weak positive allosteric modulator at higher concentrations [25].

Further research investigating the effect of variable midazolam doses on postsurgical pain in different anxiety populations might clarify our results.

### Limitations

Concerning patient recruitment, one of the units stopped enrollment due to SARS-CoV-2 pandemic restrictions. Although we enrolled a total of 296 patients, a more even distribution across units would allow for more robust external validity of the results. The low education level of our sample, particularly among the elderly, might have contributed to missing data in anxiety and pain questions; some patients needed help to complete questionnaires, which could, to some extent, influence their responses. A total of 14 patients had incomplete follow-ups, meaning that they did not answer all three follow-up telephone contacts, though all available data were included in each analysis. Concerning outcomes, we observed that analgesic doses were not consistently related to pain scores but instead were mainly dependent on routines and usual practice of each unit; thus, we decided not to use analgesic consumption as a surrogate for pain and to include the ambulatory unit as a variable in all regression models. Finally, we did not evaluate depression, pain catastrophizing, or trait anxiety, as this would increase the length and complexity of the questionnaire. However, measured state anxiety was similar to state anxiety in other populations [20]. Acute and chronic pain values were also comparable [4,18,23].

Including patients submitted to either spinal or general anesthesia under variable doses of midazolam (0–5 mg), from three different units, might have increased the risk of bias and confounding. The type of anesthesia and ambulatory unit are external factors that could simultaneously influence midazolam dose and postsurgical pain through minor variations in the surgical technique; on the other hand, a multicentre study with different populations might result in more generalizable results. Being an observational study, the lack of a formal control group (although we observed a few patients with zero dose of midazolam) and a strict single anesthetic protocol was counterbalanced with thorough multivariable regression models, analyzing midazolam as a continuous variable and adjusting for both the type of anesthesia and ambulatory unit. Interestingly, midazolam dose did not relate to preoperative anxiety (attending anesthesiologists did not have access to the preoperative questionnaire). Previous authors have shown that anesthesiologists do not correctly evaluate patient preoperative anxiety [26,27]. Therefore, our results probably reflect daily practice, where sedative premedication represents a routine intervention rather than being individualized for each patient and their level of preoperative anxiety.

We know that a randomized controlled trial (RCT) could decrease bias and better show a cause–effect relationship between midazolam and pain. However, we opted for an observational study because, in our setting, this was the most feasible to carry through. Midazolam is in such common use and, being a prospective study, we tried to measure all confounding variables to capture results in real clinical scenarios.

## 5. Conclusions

Our results uncover an effect modification of midazolam on postsurgical pain, depending on patient preoperative anxiety levels. Midazolam does not seem to attenuate the well-known hyperalgesic effect of high anxiety on postsurgical pain; paradoxically, it might be analgesic in low-anxiety patients and hyperalgesic in the very anxious ones. These results raise a new issue regarding perioperative care: midazolam, generally used as an anxiolytic, seems to only benefit the low-anxiety patients. We recommend that anesthesiologists actively measure preoperative anxiety and consider alternative strategies to reduce it, as midazolam seems to neither consistently reduce pain nor anxiety in ambulatory surgery. Further research should focus on the effect of variable midazolam doses on postsurgical pain in different anxiety populations, preferably with an RCT.

## Figures and Tables

**Table 1:** Patient characteristics and mean midazolam doses for each group.

	*n* = 281 *n* (%)		Midazolam μg.Kg^−1^	*p*	Missing Cases *n* (%)
Age (years)	<48	65 (23%)	20.3 (12.5)	0.078 ^&^	0 (0)
	49–66	151 (54%)	25.1 (14.7)		
	>67	65 (23%)	24.2 (15.2)		
BMI (Kg.m^−2^)	<26	126 (46%)	23.7 (15.0)	0.913 ^#^	4 (1)
	≥26	151 (54%)	23.9 (14.0)		
Sex	Male	250 (89%)	24.2 (14.6)	0.171 ^#^	0 (0)
	Female	31 (11%)	20.4 (12.5)		
Smoker	Yes	51 (18%)	24.6 (13.7)	0.655 ^#^	0 (0)
	No	230 (82%)	23.6 (14.6)		
Chronic benzodiazepine	Yes	20 (7%)	26.7 (14.0)	0.347 ^#^	0 (0)
	No	261 (93%)	23.5 (14.5)		
Education	<5 years	155 (55%)	24.9 (14.6)	0.270 ^&^	0 (0)
	5–9 years	57 (20%)	23.2 (13.8)		
	>9 years	69 (25%)	21.6 (14.4)		
Working status	Active	163(58%)	24.0 (13.9)	0.925 ^#^	2 (1)
	Not active	116 (42%)	23.7 (15.1)		
Preoperative pain	<4	216 (77%)	23.9 (14.6)	0.938 ^#^	1 (0)
	≥4	64 (23%)	23.7 (13.9)		
Anxiety SFQ	0–12	87 (31%)	23.0 (15.0)	0.334 ^&^	6 (2)
	13–27	98 (35%)	22.7 (14.3)		
	28–80	90 (32%)	25.6 (13.7)		
Anesthesia	Spinal	136 (49%)	32.0 (14.5)	<0.001 ^#^	1 (0)
	General	144 (51%)	15.9 (9.1)		
Surgery	RR	242 (88%)	23.5 (14.5)	0.245 ^#^	7 (2)
	Lichtenstein	32 (12%)	26.7 (13.2)		
Ambulatory unit	1	90 (32%)	16.6 (7.1)	<0.001 ^&^	0 (0)
	2	126 (45%)	27.3 (14.1)		
	3	65 (23%)	26.5 (18.6)		

Data are presented as *n* (%) or mean (SD). SFQ, Surgical Fear Questionnaire; RR, Rutkow–Robbins. ^#^ Student’s *t*-test. ^&^ One-way ANOVA.

**Table 2:** Follow-up data.

		Total *n* = 281	Midazolam μg.Kg^−1^	*p* ^#^	Missing Cases *n* (%)
At 24 h					
Pain at REST		2.2 (1.9)			20 (7)
MOV pain		4.5 (2.3)			20 (7)
Average pain		3.9 (2.0)			4 (1)
Acetaminophen (g)		1.7 (1.0)			4 (1)
Ibuprofen (g)		0.7 (0.4)			4 (1)
Rescue analgesia	Yes	30 (11%)	20.1 (7.7)	0.121	6 (2)
	No	245 (89%)	24.4 (15.0)		
Pain	<4	91 (33%)	24.4 (14.6)	0.726	4 (1)
	≥4	186 (67%)	23.7 (14.3)		
At 7 days					
Pain at REST		1 [2]			17 (6)
MOV pain		2.9 (1.8)			17 (6)
Average pain		3.3 (1.7)			9 (3)
Acetaminophen (g)		10.1 (6.8)			11 (4)
Ibuprofen (g)		4.3 (2.3)			11 (4)
Ongoing analgesia	Yes	59 (22%)	21.0 (12.3)	0.089	9 (3)
	No	213 (78%)	24.6 (14.8)		
Pain	<4	125 (46%)	24.2 (14.7)	0.731	7 (2)
	≥4	149 (54%)	23.5 (14.1)		
Satisfaction		10 [2]			8 (3)
At 3 months					
Pain at REST		0 [0]			7 (2)
MOV pain		1 [3]			9 (3)
Average pain		0 [2]			8 (3)
CPSP	Yes	72 (27%)	22.8 (14.0)	0.424	9 (3)
	No	200 (73%)	24.3 (14.3)		
Analgesic use	Yes	10 (4%)	24.6 (17.0)	0.859	9 (3)
	No	262 (96%)	23.7 (14.2)		
Satisfaction		10 [1]			7 (2)
GRSI		95 [20]			7 (2)

Values are *n* (%) or mean (SD). CPSP, Chronic Postsurgical Pain; GSRI, Global Surgery Recovery Index; MOV pain, movement-evoked pain. ^#^ Student’s *t*-test.

**Table 3:** Logistic regression models for the effect of midazolam on postsurgical pain.

Midazolam (mg)	Crude OR (95% CI)	Adjusted OR (95% CI)		
	Total sample	Total sample	SFQ 0–12	SFQ 28–80
24 h pain ≥ 4 *	0.656 (0.494–0.873)	0.630 (0.427–0.930) ^#,1^	0.407 (0.144–1.153)	0.753 (0.379–1.496)
7 d pain ≥ 1 *	0.990 (0.620–1.581)	0.418 (0.175–0.997) ^#,2^	0.261 (0.072–0.953)	0.258 (0.003–20.016)
7 d pain ≥ 4 *	0.882 (0.690–1.127)	0.764 (0.547–1.068) ^#,3^	0.289 (0.117–0.713)	0.955 (0.543–1.679)
3 mo CPSP ^&^	0.925 (0.716–1.196)	1.046 (0.742–1.474) ^#,4^	0.828 (0.415–1.654)	2.124 (1.026–4.397)

OR, odds ratio (95% confidence interval); h, hour; d, day; mo, month; SFQ, Surgical Fear Questionnaire; CPSP, Chronic Postsurgical Pain. * adjusted for sex, age, type of anesthesia, ambulatory unit, preoperative pain, anxiety SFQ, literacy, and surgical technique. ^#^ interaction between midazolam x anxiety ^1^ *p* = 0.196, ^2^ *p* = 0.071, ^3^ *p* = 0.002, ^4^ *p* = 0.295. ^&^ adjusted for sex, age, type of anesthesia, ambulatory unit, preoperative pain, anxiety SFQ, literacy, surgical technique, working status, and BMI scores.

## Data Availability

The data presented in this study are available on request from the corresponding author. The data are not publicly available due to unpublished PhD work related to the same data.
